# Improving Service Management in the Internet of Things

**DOI:** 10.3390/s120911888

**Published:** 2012-08-29

**Authors:** Chiara Sammarco, Antonio Iera

**Affiliations:** A.R.T.S. Laboratory, Department DIMET, University of Reggio Calabria, Loc. Feo di Vito, 89100 Reggio Calabria, Italy; E-Mail: chiara.sammarco@unirc.it

**Keywords:** Internet of Things, resource constrained sensors, 6LoWPAN

## Abstract

In the Internet of Things (IoT) research arena, many efforts are devoted to adapt the existing IP standards to emerging IoT nodes. This is the direction followed by three Internet Engineering Task Force (IETF) Working Groups, which paved the way for research on IP-based constrained networks. Through a simplification of the whole TCP/IP stack, resource constrained nodes become direct interlocutors of application level entities in every point of the network. In this paper we analyze some side effects of this solution, when in the presence of large amounts of data to transmit. In particular, we conduct a performance analysis of the *Constrained Application Protocol* (CoAP), a widely accepted web transfer protocol for the Internet of Things, and propose a service management enhancement that improves the exploitation of the network and node resources. This is specifically thought for constrained nodes in the abovementioned conditions and proves to be able to significantly improve the node energetic performance when in the presence of large resource representations (hence, large data transmissions).

## 1. Introduction

In the Internet of Things (IoT) scenario, strong and deep investigations have been carried out to define a unique global standard that can allow objects to communicate with each other through the Internet, despite their diversity in technologies and capabilities. Different approaches have been investigated [1–8]. Major contributions in this direction has been given by the IETF Standardization Group which started three Working Groups (WG) that basically propose to use IPv6 for object-related operations [9]. In particular, the *IPv6 over Low power Wireless Personal Area Networks* (6LoWPAN) WG [10] defined encapsulation and header compression mechanisms that allow IPv6 packets to be sent to and received from over IEEE 802.15.4 based networks. The *Routing Over Low power and Lossy networks* (ROLL) WG [11], instead, is specifying a new routing protocol for IP smart object networks. Finally, the *Constrained RESTful Environments* (CoRE) WG [12] is devoted to the definition of a framework for resource-oriented applications intended to run on constrained IP networks. The overall logic is quite linear: the knowledge already acquired for the world-wide Internet deployment is exploited and Internet pre-existing structures are adapted to the needs of constrained nodes [13,14].

This paper, in line with the IETF logic, introduces a solution for further reducing the constrained nodes' resource exploitation in terms of energy. According to the adopted IoT vision, services are the main actors. In the *traditional Internet*, nodes at the lowest levels of the network hierarchy were basically clients accessing services run by remote server. The advent of IoT pushed towards a vision of “leaf” nodes as *servers* to all intents and purposes, since they deliver services. Therefore, an accurate *service management* design is required to fulfill three IoT fundamental tasks: *autonomy, scalability*, and *adaptability*.

The contributions that mostly come near to a service management approach are those of the CORE WG, which defined the *Constrained Application Protocol* (CoAP), a specialized web transfer protocol to use with constrained networks and nodes for Machine-to-Machine (M2M) applications. *Service Management* deals with those procedures which allow the access, the advertising, the discovery, and the configuration of services in a network. In any of these cases, a packet exchange between a client (the requestor) and a server has to take place. This exchange may either happen among peer entities or by means of an intermediate node which works as a proxy. CoAP proposes a *Representational state transfer* (RESTful) solution where this exchange takes place among two peer entities at the application level. The present study stems from the observation of some critical issues associated to the CoAP behavior in terms of *fragmentation* and from their consequences on the constrained nodes' performance. The proposed approach (complementary to CoAP) suggests to keep communication at a lower level (just up to the *Network* level) to access the most constrained nodes' services, while leaving more powerful nodes to rely on application-level communication. Throughout the paper we will show that our proposal leads to energy improvements as well as to a more appealing way to address constrained nodes' services.

In more detail, the focus is on the CoAP interaction model which is based on the existing *HyperText Transfer Protocol* (HTTP) methods (GET, PUT, POST and DELETE). The intended contributions of the paper are:

- performance analysis and comparison of the fragmentation dynamics of two standard CoAP existing solutions;
- proposal of a service-oriented exchange model that could substitute CoAP *under certain conditions*;
- performance assessment of the proposed method.

The paper is organized as follows: Section 2 introduces the research problem. Section 3 describes the proposal and compares it to a standard approach based on CoAP. Section 4 presents a performance evaluation and comparison activity. Section 5 discusses rising issues related to the proposal. Section 6 overviews some related works. Section 7 synthesizes the conclusions. Section 8 sketches some planned future researches.

## 2. Research Problem Statement

**Figure 1.**
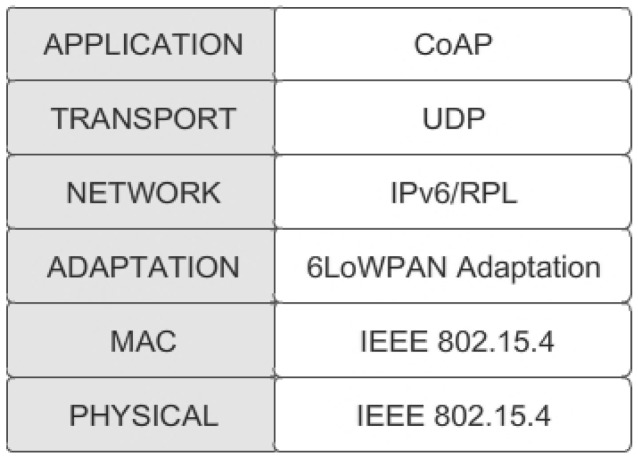
Object Protocol Stack.

Let us focus on the case where a user tries to access a node service by implementing the CoAP method and the server has to send the client back a *large* resource representation. In the considered protocol stack (see Figure 1), IEEE 802.15.4 covers the physical and MAC layers. Immediately above, the 6LoWPAN adaptation layer makes constrained nodes able to support IPv6 and it is responsible for handling IPv6 header compression, fragmentation, and reassembly. The transport protocol is UDP.

By considering that IEEE 802.15.4 MAC layer leaves just 81B frame space to the upper layers, from the viewpoint of a constrained node, two events may occur:

- fragmentation;
- message rejection because of too small a buffer.

In the *first* case, the client-server exchange takes place and the packet at IPv6 level is encapsulated into one (or just a few) IPv6 datagrams. Notwithstanding, the 6LoWPAN adaptation layer processes the information and fragments it into a number *p* of frames. Thus, the number of bytes the constrained node exchanges during the CoAP packet transmission, proportionally depends on *p*. This means that handling a large packet correspond to overload the 6LoWPAN nodes, which have to process a larger amount of bytes with respect to IPv6 nodes. Moreover, until the node has not processed all fragments, it cannot serve any other request.

In the *second* case, the node is not even able to receive the CoAP packet, since the buffer it can allocate is not large enough to contain it all. To deal with this critical issue, CoAP authors simply introduce the 4.13 response code (*Request Entity Too Large*) which should be sent by the server to tell the client that the packet has been truncated.

In order to solve the above mentioned problems, the “*Blockwise transfer*” mode has been introduced in the literature [15]. This is a new kind of data transfer for CoAP which implements a sort of application-level fragmentation that splits a single REST operation into multiple CoAP message exchanges. In conclusion, the amount of data that in the previous case was sent by means of a single CoAP packet, it is now sent through several CoAP packets. Despite this method solves the packet rejection problem, the added overhead further degrades the performance.

In conclusion, in both cases constrained nodes have to deal with an increase in the overhead in terms of bytes sent. In the remaining part of the paper we will show how this corresponds to an increase in the energy consumption, which obviously reduces the battery lifetime. Even though in the next future constrained nodes will be less constrained thanks to extra memory and more powerful processors, the question we should reflect on is: *is this the wisest way to exploit the nodes and the network resources?* The analyses in this paper aim at providing more elements to answer this question.

Another open issue is related to the definition of the resource representation used in CoAP to identify the services of the constrained nodes. Being this left to manufacturers, two objects that deliver the same services will potentially advertise them differently. Thus, programmers who want to write applications for the IoT will need to first discover the resource representation format and semantic [16]. This also means that the same application will not work for objects of different manufactures as, otherwise, an intermediate entity should solve the semantics “mismatches” in between. Our proposed solution is based on the introduction of a global unique identifier for the service type. If on the one hand this would let constrained nodes to save their energy and computational resources (as it will be shown in the next sections), on the other hand this helps the IoT to evolve in a more “*unifying*” *fashion*.

Furthermore, the solution is intended to be fully *compatible* with the existing CoAP standard because the service identifier can *easily be incorporated into the CoAP resource representation format* within one packet option. In the reference scenario, devices are classified according to their capabilities into different *levels* and during the network startup they get to know each other (IP address, device level, service IDs). Therefore, once a node knows that it is going to send a service request to a device belonging to the class of the most constrained ones, then it can switch to the communication protocol proposed in this paper, leaving unchanged the upper layer exchange for more performing nodes. This means that the proposed solution is not alternative but complementary to CoaP. In Section 5 we will further discuss some raising open issues by referring to a real world scenario in light of the analysis of the following sections.

## 3. The Proposed Approach

The proposed solution aims at reducing the amount of data, which constrained nodes have to exchange. It leans on an essential service characterization model (Section 3.1) whose parameters are the fields of the packet exploited for service management operations (Section 3.2).

### 3.1. Service Characterization Model

**Figure 2.**
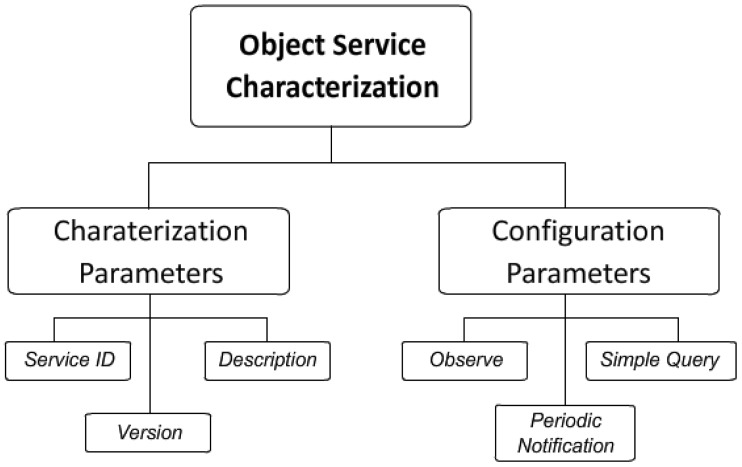
Service Characterization.

Two kinds of parameters are distinguished (Figure 2): (i) *Service Description Parameters* that describe the service (Service ID, Version, Description); (ii) *Service Configuration Parameters* that describe the service delivery (Observe, Periodic Notification, Simple Query).

The service type identification is basically implemented by the ***service description parameters***: *Version, Service ID*, and *Description* (which is optional). The *Service ID* is the most important one and it is mandatory. We conceived it as a globally-valid identifier for the type of service. In the CORE WG, several attempts to define a *resource identifier* [17] have been made but none in the direction of a *binary* globally-valid identifier. Basically, discussions focused on semantic identifiers spelled the same way as URL or URN. Since the highest contribution to the packet sizes is given by the URI resource representation, we believe that a cooperative effort (of the research community together with vendors and manufacturers) to define a globally-recognized IANA numeration for objects' Service ID would greatly help in reducing the packet size. This would enhance both the network and node performance. Moreover, it is important to keep only machine-readable information for constrained nodes' exchanges. A human-readable information (like a resource representation), indeed, is not needed for the node functioning; it only adds a packet overhead and increases the energy and bandwidth consumption.

For the Service ID, 5 bytes have been left for IANA numeration specification. However, this length may change according to the version of the IANA numeration in use, which is specified in the *Version* field. The *Description* parameter has been added for dealing with the case of those objects not yet included in the numeration (*i.e.*, for storing either *Universal Product Code* or *European Article Number* codes). In Section 5 more space is dedicated to discussions on the service identification issue.

As regards the ***service configuration parameters***, these are basically three: observe *obs*; periodic notification *pn*, and simple query *sq*. All of them are flag parameters that are used to let another node subscribe a service with a certain delivery method. In particular, the *observe method* can be used to receive a notification about changes in the state of a monitored resource. The *periodic notification* method can be used to request periodic notifications; numeric parameters (x, y, z) to define the preferred periodicity (for space constraint reasons, details are avoided). For the *simple query* case, setting the *sq* flag to 0 or 1 means “enable with authorization” or “enable”, respectively. The *simple query* is the basic service; hence, restricting the access to it implies restricting the access to all other service delivery options. If the *sq* flag is set to 0, then an authorization code will be required to access that particular service in any modality. The authentication issue is out of the scope of this paper, thus it is not addressed.

### 3.2. Packet Size Evaluation and Comparisons

To implement our solution, we introduce a *Service Management* (SM) packet which encodes three kinds of information: (i) type of operation; (ii) service identification information; (iii) service delivery method. Table 1 shows the size of all fields.

Each of them corresponds to a parameter of the service characterization in Section 3.1, but the following:

- *Type:* identifies the type of message (*Confirmable, Non Confirmable, Acknowledgement, Acknowledgement plus data*);
- *Code:* specifies the kind of method to query a service (*i.e.*, to access it, to modify the subscription, to unsubscribe). In case of response, it contains a response code;
- *Message ID:* detects duplicates and matches confirmable or reset messages with their acknowledgement;
- *Length:* length of the packet.

The SM packet is implemented as an ICMPv6-like packet. Both description and performance analysis of packet retransmission, loss handing, duplicate detection are out of the scope of this paper even if *Message Identifier* and *Type* fields have been thought to support future mechanisms addressing those problems.

In the remainder of this section we compare our proposal, which has a *service*-oriented nature, to the two CoAP solutions proposed in the IETF CORE WG and described in Section 2. Since these solutions are resource-oriented, an extra attention will be paid while comparing.

A CoAP message consists of a header followed by options in *Type-Length-Value* (TLV) format and a payload [18]. Only the header size is fixed at 4 bytes, the other two fields are optional and of a variable length. In official IETF documents, the authors suggest developers to be frugal in the packet size choice for their applications since it may cause a very important fragmentation phenomenon, especially at the adaptation level (6LoWPAN messages are limited to 127 bytes including various overheads). For this reason, the CoAP authors claim that “*a CoAP message, appropriately encapsulated, should fit within a single IP packet*” (intending IPv6 datagram which has an MTU of 1,280 bytes); thus defining a kind of generic upper layer.

**Figure 3.**
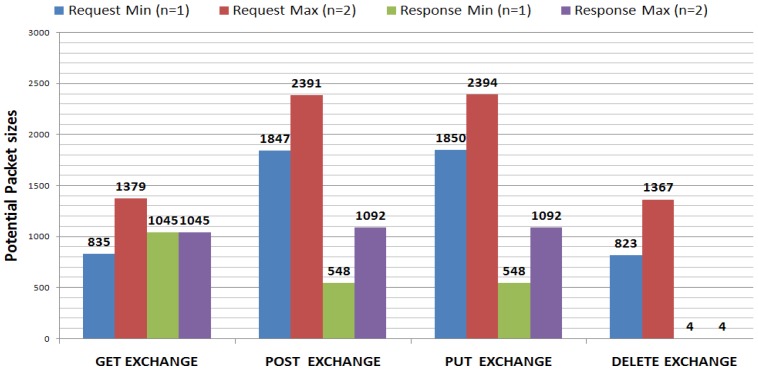
Potential Maximum CoAP Packet sizes.

Figure 3 shows the potential maximum values of the CoAP packet size. It is obtained by considering the information given in [18], related to the information (options, payload) contained in each packet according to the used method (GET, PUT, POST, DELETE) associated to a *resource*. For each interaction, a distinction is made between the CoAP packet sizes of a request and of a response. We assumed the maximum length for the options and 1,024 bytes for the payload size (this value is not fixed since it depends on the resource representation length). The *n* parameter in the labels refers to the fact that some options may appear *one or more* times in the same packet. In order to keep CoAP packet size near to the suggested maximum size, *n* in this case has to assume very low values (*n* = 1, 2).

From Figure 3 it emerges that the most byte-expensive methods are PUT and POST and that, globally speaking and considering *n* equal to 2, the sizes of the CoAP packet are kept under the threshold of 2.4 kbytes. Basically, the packet size variations depend on the variation in size of the URI resource representation that occupies—except for the 4B header—the entire packet.

While CoAP uses the GET, POST, PUT, and DELETE methods associated to a *resource*, the proposed model, instead, assumes the following procedures: *accessing* a service (associable to a GET request), *subscribing* to it (associable to a POST exchange), *modifying* the way of delivery (associable to the PUT exchange) or *unsubscribing* (DELETE method).

**Figure 4.**
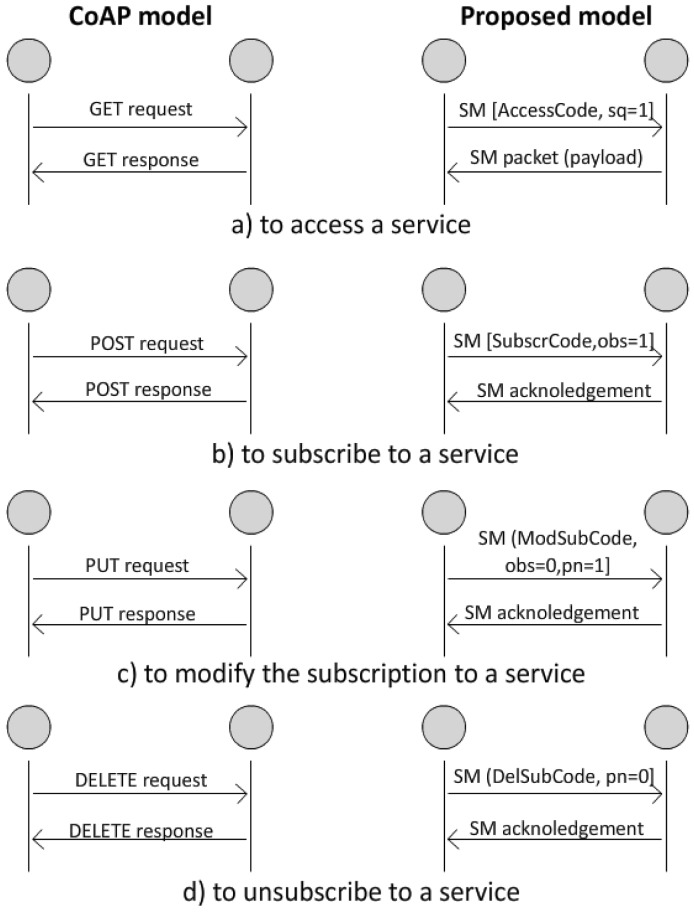
Matching among exchange methods.

Figure 4 shows a comparison between the logical exchanges in the two cases. To differentiate the SM packet, the *Code* field is used, while the *Type* field indicates a Confirmable packet in all of the cases shown in the figure. For each transaction, except for the *access* case, the proposed model foresees a response that is a simple acknowledgement.

In the *access* case, the *sq* flag is up in the request packet. The response is of the *acknowledgement plus data* type and contains a payload. The payload may contain the sensed value (in case of *sensing* service), a Boolean (in case of simple actuators), or the RFID tag content (in case of *identification* service). In case of special smart objects, it may also contain more complex data according to vendor specifications but, in any case, the payload does not contain any descriptive text.

To optimize the lower layer payload usage, it is desirable that the SM packet size fills the reserved space (81 bytes) in the IEEE 802.15.4 frame. The *Service ID* code may be used as an index to check the output format of the devices (from manufacturer online-available registry). Anyway, if one considers that mandatory fields are 10bytes-long according to the above mentioned specifications, then the manufacturers should try to limit the payload size (and hence the device returned values) up to 71 bytes.

Table 2 shows the total number of IEEE 802.15.4 frames and bytes sent for each transaction according to the proposed solution. The calculation includes: (i) for each packet, the fields in Table 1 along with the 6LoWPAN header; (ii) for each transaction, the request and the response; (iii) a 71 bytes payload for the *access* case.

In conclusion, from all the considerations in this section it emerges that the use of the Service ID code helps to considerably reduce the packet size because it identifies the constrained node's service without having to describe it (as the *URI resource representation* does in the CoAP case). As a consequence, the fragmentation and rejection problems get solve for the case that have been illustrated in Section 2 (*large resource representation*).

## 4. Analysis and Performance Comparison

The first part of this section analyses the performance of the CoAP protocol on 6LoWPAN nodes. The study aimed at conducting a worst case analysis on the effects that large resource representation transfers in CoAP have onto the lower layers in terms of fragmentation and rejection risk (a relevant issue within the CORE WG [15]). Subsequently, we illustrate simulative results that compare the CoAP model with the proposed model in terms of energy consumption; this demonstrating the higher performance of the latter.

### 4.1. CoAP Packet Size Effects on Lower Levels

Let us consider both cases in which an IPv6 node and a 6LoWPAN node process the packet. Two hypotheses are valid: (i) no losses; (ii) no restrictions for the buffers size.

For the calculation, we consider 48 bytes per packet overhead for the IPv6 node, since UDP is the transport protocol. For the 6LoWPAN case, after the adaptation, this information is compressed into fewer bytes (from 6 to 11 bytes). Moreover, while in the IPv6 case we can rely on 1,280 bytes of MTU, in the 6loWPAN case just 81 bytes of the 127 bytes of the IEEE 802.15.4 frame can be used by the upper layers (by assuming a secure transmission with AES-CCM-128 encryption). The remaining bytes of the IEEE 802.15.4 frame are used for both the MAC layer exigencies and for the compressed UDP and IPv6 headers.

**Figure 5.**
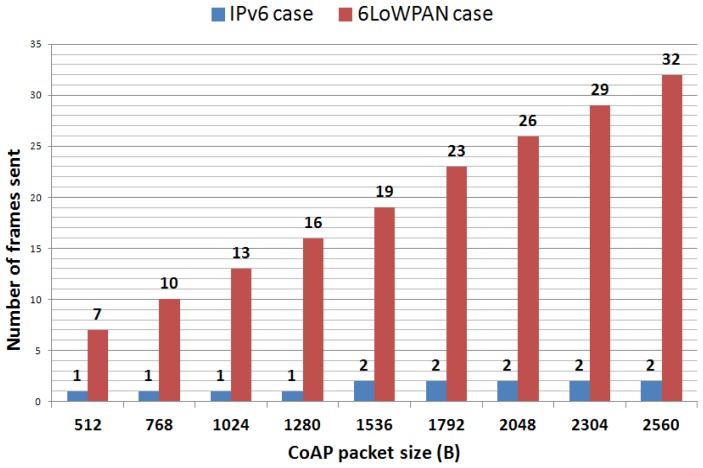
Number of packets sent as a function of the CoAP packet size.

**Figure 6.**
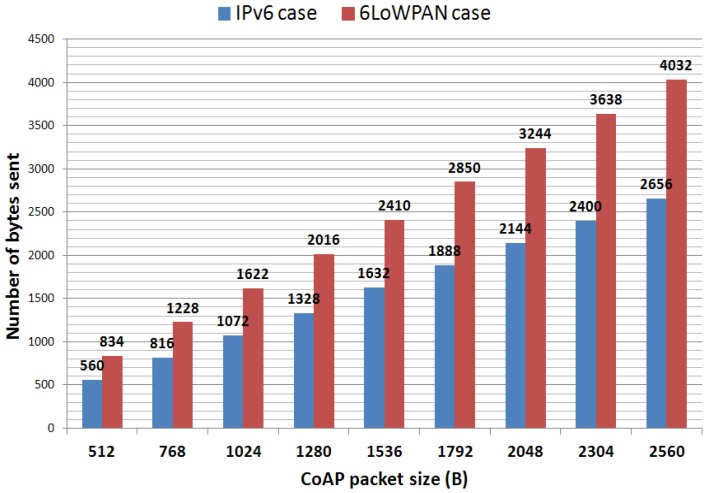
Number of bytes sent as a function of the CoAP packet size.

Figures 5 and 6 respectively show the trends in the number of IEEE 802.15.4 frames and bytes sent as a function of the CoAP packet sizes (chosen according to the packet sizes described in the previous section). As expected, the larger the packet size, the higher are both the number of IEEE 802.15.4 frames sent and the global overhead added to the CoAP packet (with respect to IPv6). This analysis helps to figure out the great disparity in the treatment of IPv6 and 6LoWPAN nodes. The most constrained ones (6LoWPAN) would have to process nearly 50% extra bytes with respect to the most efficient ones (IPv6).

Finally, Table 3 reports the same values on a transaction basis, according to the CoAP method in use. Non-confirmable request and response messages are considered.

Let us observe what happens when activating the *Blockwise transfer* mode to guarantee the CoAP packet transmission. This is a new kind of data transfer for CoAP that splits a single REST operation into multiple CoAP message exchanges, by exploiting two new CoAP options. For major details, please refer to [15]. The main objective of the Blockwise transfer mode is to avoid creating a *conversation state* at the server. This allows a transfer made on a block-basis; for each block two messages are exchanged (*i.e.*, in the GET case, one packet is sent to request the *i*-th block and the other one is sent back with the requested information). Results of this analysis are obtained by assuming *ideal RF conditions* (*i.e.*, no losses) and real buffer sizes.

Table 4 shows the number of IEEE 802.15.4 frames and bytes sent on a transaction basis, according to the CoAP method in use and assuming the potential maximum CoAP packet size shown in Figure 3. For the computation, a block size of 128 bytes is chosen (enough to use a 1byte-block option, since it has a 4-bit NUM field which allows to address 16 packets).

The adoption of this transfer mode implies:

- In case of a GET request, the client has to send the same CoAP request with all the options *nb* times (with *nb* equal to the number of blocks the server needs to send back the resource representation). This causes a waste of the server node computational resources (same request processed *nb* times) and a relevant increase in the number of bytes sent (first line of Table 4).
- The possible sizes of the blocks do not fit the 6LoWPAN packet payload (81 bytes); thus, the payload space is wasted as many traveling frames are nearly empty.

**Figure 7.**
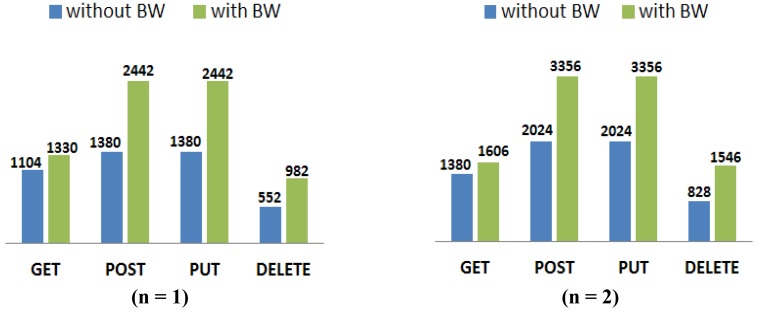
Added overhead *(BW* stands *for Block-Wise)*.

In conclusion, the use of the *Blockwise transfer* mode solves the *packet rejection* problem and avoids creating a conversation state on the server by distributing the load on a larger transmission and by serving more requests simultaneously. On the other hand, *it does not solve the fragmentation issue but just migrates it to upper layers*. Moreover, the overall load for the same transaction (GET, POST, PUT, or DELETE) increases, as a comparison between values in Tables 3 and 4 shows. Finally, the total added overhead (difference between bytes sent by 6LoWPAN and CoAP packet sizes) is definitely higher in the *Blockwise* (BW) case, as shown in Figure 7.

### 4.2. Performance Evaluations in Terms of Energy Waste

This section illustrates a simulative performance comparison between the proposed solution and the CoAP one, obtained by means of the COOJA simulator for the Contiki Operating System [19]. In both cases, nodes emulate the Tmote Sky platform and implement the 6LoWPAN adaptation layer. The node behavior has been observed for the ContikiMAC [20] RDC (*Radio Duty Cicling*) layer mechanism, which is the default one in Contiki OS. The solution embedded in COOJA and described in [21] has been used for the energy estimation. During the simulation, a client node asks for a service to a server node with a periodicity of 10 seconds. In particular:

- for the *proposed model, Node 1* sends Service Management (SM) packets to *Node 2* to access a *sensing* service and it obtains a numerical value back
- for the *CoAP model*, the simulation environment consists of two nodes: a *REST Server* and a *CoAP client* (we used the CoAP implementation presented in [22]). The analysis is differentiated for four different service requests: light sensor, battery level, toggle and resource discovery

Energy measurements, performed with a periodicity of one second, last for 300,000 ms and have been differentiated for the following components: *reception* (Rx), *transmission* (Tx), *processing* (CPU), and *low-power-mode* (LPM). The tables in this subsection (Tables 5 and 6) will show the *total* energy consumption during the entire simulation interval. Also the results relevant to the *percentage distribution* in Figure 9 refers to the illustrated simulation setup.

As previously observed, the CoAP packet size may reach the potential maximum shown in Figure 3. Whilst, the proposed model, by using the Service ID code, does not make the service representation increase. Thus, we argue that for large resource representation transmissions, the proposed model may help in increasing the performance.

Tables 5 and 6 show the energy consumption values (in mJ) respectively for the introduced SM transmission and for the CoAP packet transmission. One can observe that for short resource representations the SM packet usage does not lead to any visible improvements.

**Figure 8.**
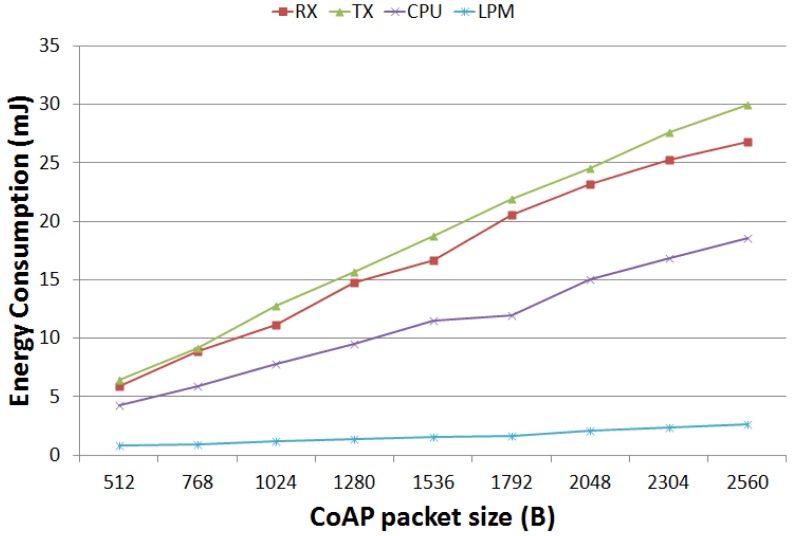
Energy Consumption as a function of the CoAP Packet Size.

The “Discover” column in Table 6 gives information on what happens when larger resource representations are sent. The energy consumption trend is better investigated in Figure 8, for gradual increments of the CoAP packet size. In the relevant simulation setup, differently from above, a server periodically sends to a client a CoAP packet of the size shown in the *x* coordinate. The figure reports the *average energy* values on the server side differentiated for each energy components. The analysis shows a significant dependency of the *transmission* (Tx), *processing* (CPU), and *reception* (Rx) components from the amount of bytes sent. Whilst, the LPM component slope is much less significant.

**Figure 9.**
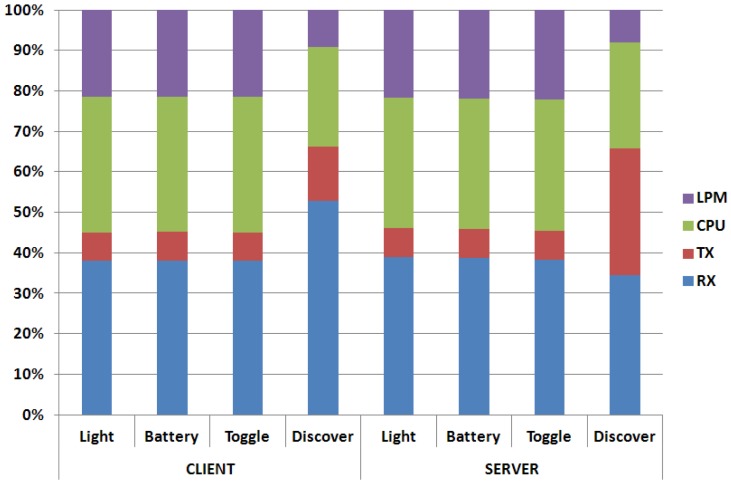
Energy Consumption percentage distribution among the reception (RX), transmission (TX), processing (CPU), low-power-mode (LPM) components for different CoAP exchanges (ContikiMAC case).

Figure 9, instead, shows the percentage energy distribution among all the components. By looking at these results, one can notice that the *reception* (Rx) component is responsible for the highest energy cost, followed by the processing (CPU) one. For a larger data forwarding (*i.e.*, the server in the discovery case), the transmission (Tx) component assumes comparable values.

## 5. Final Remarks

As shown in the previous sections, the proposed solutions leads to an improvement of the constrained nodes performance for large resource transmissions. In this section, we will discuss important arising issues to allow the reader to better understand the benefits, functional behaviour, and potentials of our proposal. The solution is based on the service characterization model described in Section 3, in which the Service Identifier plays a key role. Therefore, we first focus on the capability of the proposed system to address both existing and future services (Section 5.1). Afterwards, in Section 5.2, considering that the proposed solution will have to coexist with existing (CoAP) standards, we further address compatibility issues by describing a sample real world scenario.

### 5.1. Addressing Space and Scalability

In this section we are interested in answering the following questions: (i) How can we be sure that the definition of the Service ID, together with the fields in Table 1, will allow to address all the existing services? (ii) When considering new objects (and hence services) that may be created in the future, how will this solution incorporate them and adapt to any other future scenario?

Since an exact *a priori* estimation of the number of service types that will characterize the future IoT is infeasible, we designed our service characterization model so to make its encoding mechanism flexible and capable of enabling future extensions. What we propose is a progressive migration to a IANA numeration scheme that allows the inclusion of new services thanks to the following model attributes:

- the *Version* field that identifies the version of the IANA numeration in use
- the *Description* optional field, thought as an identifier of those services not yet included in the IANA numeration. For example, in the case of a new actuator (not yet included in the IANA numeration), the Description field may be used to store an object-related identifying information such as *Universal Product Code* (UPC) or *European Article Number* (EAN)

To show the benefits of defining a compact and fixed service type encoding mechanism, the estimation of a Service ID-field size that could fit a worst case analysis is required. As regards the capacity of a 5B-long code to address the future *Service ID*-space expansion, a first indication comes from the Moore's and the Metcalfe's Laws. In fact, when considered together, they mean that continued network expansion is inevitable. However, they predict an increase in the number of *nodes* not in the number of *node types*. Past experiences in Telecommunications tell us that the latter number is usually much smaller and it also increases much more slowly. In conclusion, a 5B-long Service ID sounds as a comfortable estimation, since it allows the usage of an address space of 2^40^ elements.

It has to be noticed, that this estimation considers *the number of service types* statistically correlated to the *number of device types*. This sounds reasonable because, even if more than one service can be advertised by a certain device, the same service may belong to different devices.

Giving details on the specific IANA numeration is out of the scope of the present work. Thus, hereafter, we describe the reasoning that brought to the envisioned numeration to allow the reader to achieve a conceptual comprehension of the same.

We started by considering the kind of objects that belong to the IoT, and grouping them into three families: sensors, actuators, and identifiers (*i.e.*, RFID tags). Consequently, we looked for any existing classification of the number of device classes for each family, trying to foresee their possible future expansion.

**Figure 10.**
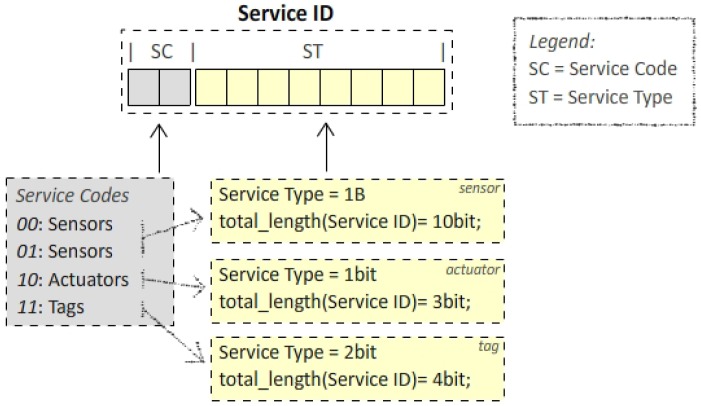
Rough Service ID classification.

For the sensor's family, we found a starting approximation for the number of elements in [23] where a list of existing sensors can be found. We saw that 1 byte was enough to address all the sensors types listed there. Anyway, as shown in Figure 10, we added 1 extra byte. In the tags' family we could just start distinguishing into active, passive, and semi-passive tags. At this beginning level, we classified the actuators into simple (just *on* or *off* state are allowed) and complex one (all the others).

The described reasoning is intended to give an idea of the kind of numeration envisaged: a binary globally-valid encoding purified from the overhead due to human-readable information (which is not needed for its functioning). By extending the length size up to 5 bytes, a more accurate characterization (especially for the actuators' family) is possible. *Application domain*-based classification could be exploited in order to make the encoding not only *tidy*, but also *smart* (*i.e.*, the applicative scenario could be encoded into a portion of the identifier, so that, for example, actuators of the same class type, but built for different contexts would just differ for a few bits). Indeed, starting from a statistical analysis, the encoding could be made shorter, for example, for highly popular services on the most constrained devices.

### 5.2. Real World Scenario

**Figure 11.**
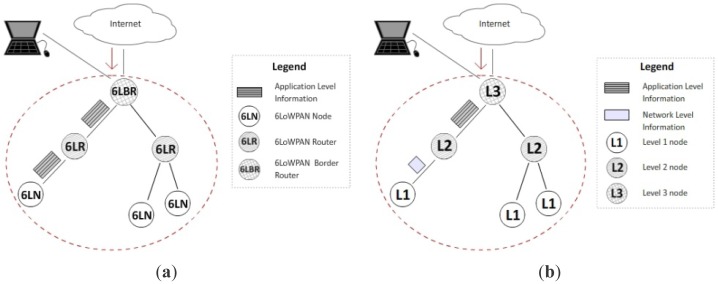
Communication models: (**a**) CoAP model; (**b**) proposed model.

Let us now focus on a real world sample environment. In the reference scenario (Figure 11(b)), devices are classified according to their capabilities into different level (Level 1, 2, and 3). During the network startup, they get to know each other (through their IP addresses, device level, and service IDs). Let us suppose that the one shown in Figure 11 is the object domain of a domotic environment for the two models under analysis. A query from the Internet reaches a leaf node (*i.e.*, sensor or any kind of house facility) of the object network for accessing one of its services passing through the root and an intermediate node.

For the CoAP model nodes (described in [24]) 6LoWPAN Node (6LN), 6LoWPAN Router (6LR), and 6LoWPAN Border Router (6LBR) are defined. All of them implement the CoAP layer and, therefore, they exchange packets (in this case a GET exchange occurs) containing application-level information (Figure 11(a)) with a representation of the accessed resource (as illustrated previously).

For the proposed model case, CoAP communication is kept among devices of Level 2 and Level 3 (Figure 11(b)), but CoAP packets have two extra options that contains the information of the *Version* and *Service ID* fields of Table 1. Once the requesting CoAP packet with the GET request reaches the Level 2 node, it switches the communication up to the *Network Level* by using the proposed SM-packet exchange illustrated in Section 3. In particular, the *request* SM-packet will be of *Confirmable* type, with the *AccessCode* in the *Code* field. The *sq* flag will be set up for indicating a simple query and the *Service ID* will identify the queried service. The response packet (*i.e.*, the one the Level 1 node sends back to Level 2) will be of *Acknowledgement plus data* type and it will contain the payload. Once the response is received, the Level 2 node will convert it back into a GET response for the Level 3 node.

The SM packet exchange is independent from the link layer technology; hence, it is *extensible to any network supporting a 6LoWPAN exchange*. Moreover, Service ID-based encoding helps to implement a uniform resource naming in the IoT since, by using the CoAP model, two objects that deliver the same services will potentially advertise them differently. This means that programmers who want to write back-end applications or middleware solutions for the IoT, by using our solution, will not need to first discover the particular resource representation of the underlying object domain, but they could just refer to any IoT service by referring to its Service ID.

It has to be noticed that, the *(IP address, Service ID)* couple uniquely identifies a certain service on a device. Basically, it gives the same information provided by the *(IP address, transport port)* couple in a standard TCP/IP network. Moreover, *Message ID* and *Type* fields of the SM packet are means to handle issues like packet losses, retransmissions and, duplication of packets whose discussion, however, is out of the scope of the present paper.

As regards the application-level information care, this is up to application-level nodes. Constrained nodes will simply return information related to their services as foreseen for the kind of services they deal with. In other words, for each kind of service (Service ID), a specific response format will have to be expected. Once the reply is obtained from constrained nodes, it will be shaped into the requested format by the first application level entity in the path.

## 6. Related Works

In the traditional Internet, web services have always been put into relationship with the World Wide Web, hence they were hidden by web pages or queried by client-side applications. Originally, web services were heavyweight entities, thus not easily transferrable to devices with high constraints. Documents [25] and [26] demonstrated that it is reasonable to associate web services also to smart objects. The authors of [25] investigated service-oriented solutions that use *eXtensible Markup Language* (XML) transactions enclosed into *Simple Object Access Protocol* (SOAP) messages sent over HTTP and TCP. In [26], the authors presented an analysis of the REST transactions over HTTP. Document [17] defined a Web Linking by using a link format for constrained web servers to describe hosted resources. Moreover, in order to realize a *CORE Resource Discovery*, a well-known *Uniform Resource Identifier* (URI)—well-known/core—is defined as a default entry-point for requesting the links hosted by a server. [27] defined a mechanism to register the notifications in case of resource state changes.

In [22], the authors also point out that: (i) not all CoAP resource representations can fit into a single IEEE 802.15.4 frame, so that either 6LoWPAN fragmentation or Blockwise transfer is required; (ii) the energy cost of Blockwise transfers would correspond to multiple requests with the payload size adjusted for the additional header option. Therefore, to enable energy-efficient transmissions of consecutive frames, the authors propose to use link-layer bursts. Basically, when a sender has several frames to send, it first wakes its neighbors up with a ContikiMAC strobe and sets the “frame pending” bit in the IEEE 802.15.4 frame header to tell the receiver that another frame will follow. Remaining frames are sent consecutively and acknowledged by the receiver until the frame pending bit is unset. This solution looks pretty interesting even if it is valid just for the IEEE 802.15.4 nodes. The solution we propose in the following sections is independent from the low layer technologies because it works at the *Network Level*.

In the literature a reasoning similar to the one proposed in this paper, which is based on *non-human-centric specification*, can be found in some M2M solutions close to the *Semantic Web* vision. In this vision, the information is machine processable and automated agents retrieve, extract, and combine it. In [28], authors explain how semantic technologies could play a key role in the Internet of Things. In particular, for their way of architecting the Web, the solutions based on *OWL Web Ontology Language* [29] (a standardized W3C technology) come very near to our proposal. Indeed, they are designed for applications that need to process the content of information, instead of just presenting information to humans. However, up to some years ago, OWL-based tools were too much complex and resource intensive for tiny nodes. In the recent years, research tried to expand *Semantic Web* techniques and tools to deal with resource-constrained devices. Document [30] reviews the state of the art for the semantic specification of sensors (one of the fundamental technologies in the *semantic sensor network* vision) which aims at solving difficulties in installing, querying and maintaining complex, heterogeneous sensor networks. In [31] authors developed an OWL *semantic reasoner* for embedded devices.

Authors of [32] and [33] present algorithms and encoding schemes for compressing representations. In particular, in [32] a coding scheme for *ontologies* that embeds semantic awareness into devices with limited memory and processing capabilities is developed. This scheme provides a compact representation of an ontology and is enhanced with an efficient and effective semantic-matching algorithm. Finally, document [33] presented and evaluated an approach that saves memory without loss of reasoning ability, which facilitates OWL reasoning on constrained devices.

## 7. Conclusions

In this paper we have introduced a solution for constrained nodes' *service management* that improves the exploitation of *network and node* resources for large resource representation transmissions. The main idea is to keep the communication for the most constrained nodes at the *Network level* so that the information is purified from transport and application data. This is done by basing the proposed solution on a very essential service characterization model, which foresees that the access to constrained node services is done by means of very short packets.

Following an analysis on the effects of the CoAP packet sizes on the lower layers in terms of fragmentation, a simulative study is conducted in a COOJA environment. It aimed at comparing the performance of the proposed approach with the traditional CoAP approach in terms of energy waste. The obtained results testified to a better performing behavior of the proposed transmission method (when compared to standard CoAP) *for large resource representations transmission.* While, for short resource transmissions it does not lead to any visible *energy* improvement. In particular, the energy benefits have been shown by means of an analysis for gradual increments of the CoAP packet size. This testified to a significant dependency of the *transmission* (Tx), *processing* (CPU), and *reception* (Rx) energy components from the amount of sent bytes. Being, indeed, the proposed model based on a Service ID code (identifying the *type of service*), this does not make the service representation increases (like for the CoAP case).

However, in any case (both *large* and *short* service representations), the proposed method has the advantage to enable a unique *global service identifying scheme*. This lets programmers, who want to write back-end applications or middleware IoT solutions, to just refer to any *IoT* service by referring to its Service ID without the need to first discover the particular resource representation of the underlying object domain. Moreover, the proposed solution is backward-compatible, scalable, and independent from the link layer technology.

## 8. Future Research Works

In Section 5.2, we mentioned a procedure for the network startup through which nodes get to know their IP addresses, device level, and service IDs. The procedure we have in mind is an extension of the *neighbor discovery* mechanism foreseen for the 6LoWPAN nodes bootstrap. Indeed, the identification of the service type with a code (Service ID) make it possible for 6LoWPAN nodes to advertise their services along with their IP addresses as soon as they get to know their default router. This would allow more performing nodes to create a distributed *network service registry* that can prevent most constrained nodes from being massively queried for service discovery procedures. Basically, the most constrained nodes would be queried only if they do offer the sought service. Future research works will be focused on evaluating drawbacks and benefits of the envisioned *service advertising* and *discovery* mechanisms.

As announced in the *research problem statement* in Section 2, the proposed solution tackles large transmission problems caused by *large resource representation*. This is in line with the focus of the paper, which focuses on the access to constrained nodes' services. However, future research works may investigate the extension of the use of the SM packet for other *large transmission* cases (*i.e.*, network management operations like *nodes' firmware update*). The logic of the link-layer burst suggested in [22] may be exploited. As already mentioned, the solution is very interesting, but it is valid just for IEEE 802.15.4 nodes. The exploitation of the SM packet would make it independent from the *low layer technologies* since it works at the Network Level.

Finally, further future research works will be finalized to enriching the envisioned *Service ID* encoding in the light of the semantic solutions for constrained nodes highlighted in Section 6. This would let the proposed mechanism evolving towards the perspective depicted in [34], where middleware solutions enhanced by semantic technologies are pointed as the most promising technologies to tackle interoperability, self-management, and autonomous issues.

## Figures and Tables

**Table 1.** Packet fields length.

**Name**	**Mandatory/Optional**	**Description**	**Length**

Type	M	Message Type	2 bit
Code	M	Kind of operation or response code	1B
Message ID	M	Message Identifier	2B
Length	M	Message Length	1B
Version	M	Version of the Service ID numeration	3 bit
Service ID	M	Identification code for the service type	5B
Description	O	Further service type identification	20B
Obs	M	Flag for the *observe* delivery method	1 bit
Pn	M	Flag for the *periodic notification* delivery method	1 bit
Sq	M	Flag for the *simple query* delivery method	1 bit
X	O	Numeric variable to set the periodicity	2B
Y	O	Numeric variable to set the periodicity	2B
Z	O	Numeric variable to set the periodicity	2B

**Table 2.** Analysis of the Proposed Solution.

		**Total number of 802.15.4-frames**	**Total number of Bytes**

**GET**	**Access**	2	203 B
**POST**	**Subscribe**	2	138 B
**PUT**	**Modify Subscription**	2	138 B
**DELETE**	**Unsubscribe**	2	132 B

**Table 3.** Analysis of CoAP on a transaction basis.

	**Total number of 802.15.4-frames**		**Total number of bytes**	

	***Min (n* = *1)***	***Max (n* = *2)***	***Min (n* = *1)***	***Max (n* = *2)***

**GET**	24	31	2,984 B	3,850 B
**POST**	30	44	3,775 B	5,507 B
**PUT**	30	44	3,778 B	5,510 B
**DELETE**	12	18	1,379 B	2,199 B

**Table 4.** Analysis of CoAP on a transaction basis; *Blockwise transfer* mode activated.

	**Total number of 802.15.4-frames**		**Total number of bytes**	

	***Min (n* = *1)***	***Max (n* = *2)***	***Min (n* = *1)***	***Max (n* = *2)***

**GET**	116	179	13,946 B	21,740 B
**POST**	51	71	4,887 B	6,935 B
**PUT**	51	71	4,890 B	6,938 B
**DELETE**	21	34	1,859 B	3,041 B

**Table 5.** SM-packet energy consumption calculation (ContikiMAC RDC layer).

	**SERVER**	**CLIENT**

**RX**	84.114 mJ	82.657 mJ
**TX**	14.235 mJ	13.952 mJ
**CPU**	74.039 mJ	72.517 mJ
**LPM**	46.748 mJ	46.694 mJ

*Total*	*219.136 mJ*	*215.82 mJ*

**Table 6.** CoAP-packet Energy Consumption calculation (ContikiMAC RDC Layer).

	**CLIENT (mJ)**				**SERVER (mJ)**			

	**Light**	**Battery**	**Toggle**	**Discover**	**Light**	**Battery**	**Toggle**	**Discover**

**RX**	82.8	82.7	82.6	262.1	84.3	83.1	81.4	193.0
**TX**	15.3	15.4	15.2	67.5	15.4	15.3	15.3	174.3
**CPU**	73.1	72.8	72.6	122.5	69.5	69.3	68.8	146.8
**LPM**	46.6	46.6	46.6	45.1	46.9	46.9	47.0	44.9

*Total*	*218.0*	*217.7*	*217.2*	*497.3*	*216.2*	*214.8*	*212.5*	*559.2*
